# Restructuring brain drain: strengthening governance and financing for health worker migration

**DOI:** 10.3402/gha.v6i0.19923

**Published:** 2013-01-15

**Authors:** Tim K. Mackey, Bryan A. Liang

**Affiliations:** 1 Institute of Health Law Studies, California Western School of Law, San Diego, CA; 2 Joint Doctoral Program in Global Health, University of California San Diego–San Diego State University, San Diego, CA; 3 Joint Program on Health Policy & Law, UC San Diego-California Western School of Law, San Diego, CA; 4 San Diego Center for Patient Safety, University of California San Diego School of Medicine, San Diego, CA; 5 Department of Anesthesiology, University of California San Diego School of Medicine, San Diego, CA

**Keywords:** brain drain, health worker migration, health policy, global health, global health governance, health system strengthening

## Abstract

**Background:**

Health worker migration from resource-poor countries to developed countries, also known as ‘‘brain drain’’, represents a serious global health crisis and a significant barrier to achieving global health equity. Resource-poor countries are unable to recruit and retain health workers for domestic health systems, resulting in inadequate health infrastructure and millions of dollars in healthcare investment losses.

**Methods:**

Using acceptable methods of policy analysis, we first assess current strategies aimed at alleviating brain drain and then propose our own global health policy based solution to address current policy limitations.

**Results:**

Although governments and private organizations have tried to address this policy challenge, brain drain continues to destabilise public health systems and their populations globally. Most importantly, lack of adequate financing and binding governance solutions continue to fail to prevent health worker brain drain.

**Conclusions:**

In response to these challenges, the establishment of a Global Health Resource Fund in conjunction with an international framework for health worker migration could create global governance for stable funding mechanisms encourage equitable migration pathways, and provide data collection that is desperately needed.

Disproportionate healthcare worker (‘HCW’) migration from resource-poor countries to high-income countries, aka ‘brain drain,’ creates access issues in communities already suffering from high infant mortality rates, low life expectancy, and the dual burden of non-communicable and infectious diseases (1, 2). The resulting impact on health outcomes in these resource-poor settings is a serious global health equity concern, with the World Health Organization (‘WHO’) estimating a shortage of some 4.3 million HCWs globally (1–4).

Health workforce imbalances away from resource-poor and to high-income countries have persisted for decades, yet the increasing dual burden of disease in resource-poor settings has hastened the need for an effective response (5, 6). Indeed, resource-poor countries provide 20% of all OECD country physicians, and add 5% to the annual increase of EU HCWs (3). Furthermore, high-income countries utilize 23–28% of all international physician graduates, with resource-poor countries contributing 40–75% of this total (7).

The striking imbalance results in North America and Europe gaining 65% of HCWs, yet bearing only 20% of global disease burden (4). Africa, in stark contrast, bears 24% of this burden with merely 3% of the global health workforce (4). In fact, of the 57 countries identified by the WHO as facing a *critical* HCW shortage (i.e. do not meet the minimum criteria of 23 HCWs/10,000 population), 36 are in Africa (4, 6). Studies that have attempted to estimate the loss in human capital investment of physician education in sub-Saharan African countries have estimated the costs to be as high as $2.17 billion, with often equally large benefits inuring to destination countries (8). Other organizations such as the International Organization for Migration estimates that developing countries incur some $500 million in lost education costs due to HCWs emigrating to other countries (1, 9).

Certain countries in southeast Asia (i.e. Cambodia, Indonesia, Laos, Myanmar and Vietnam) also suffer from WHO critical HCW shortages (10). Yet ironically, some of these southeast Asian countries have a robust trade in the health services sector that promotes foreign medical tourism, while others, such as the Philippines and Indonesia, actively engage in the export of their HCWs to other countries, yet fail to meet their own domestic health needs (10–12).

Brain drain can also occur within resource-poor countries through ‘internal migration,’ e.g. from rural to urban settings or from national health systems to donor agencies and NGOs (5, 13, 14). Increased funding for vertical global health initiatives and their NGO partners has given rise to competition from NGOs in HCW recruiting that has exacerbated internal migration away from local and public sectors to higher paying private sector NGO positions (6, 13, 15). This flight within and externally from resource-poor settings places serious strains on community-based health and core public health services such as maternal-child health services, immunizations, and other preventative care (2). With approximately 75% of physicians and 60% of nurses located in urban areas, access by half of the world's population living in rural areas is compromised by these imbalances (16).

High-income country health needs’ ‘pull’ factors, e.g. aging populations and higher wages, drive this demand, while poor working conditions, lack of resources, and scarce funding provide the push (1, 16), resulting in resource-poor countries HCW shortages in the millions (4, 16). Yet HCW migration may not always be efficient, with ‘brain waste’ (i.e. HCWs migration resulting in underuse, underemployment or unemployment of healthcare skills) leading to loss of human health resources for origin and destination countries alike (1, 11).

Collectively, these factors create imbalances in the global distribution of HCWs often within communities already suffering from the worst shortages and greatest needs, which then lose their HCWs to higher-income markets facing their own shortages (2, 10, 11, 17). To address these challenges, we first identify limitations of current strategies, and then propose a governance system that develops sustainable funding mechanisms through capacity building support and HCW retention and training programs, coupled with an enhanced immigration pathway to ensure equitable and efficient movement of HCWs.

## Challenges of current strategies

Domestic health systems strengthening (HSS) is crucial to preventing brain drain and retaining HCWs, as it can act as an enabling factor in attracting and retaining health workers and graduates. Improved conditions, fair remuneration, and education would allow HCWs in resource-poor countries to function effectively, while also providing potential long-term social benefits (5). Unfortunately, resource-poor countries’ governments are fighting against poverty and declining public health budgets, and may be limited in the ability to invest in public-sector healthcare systems due to economic policies of structural adjustment and debt obligations (11, 18, 19). The poor working conditions and limited resources in rural areas make these challenges even more difficult to surmount (5).

Consequentially, external donors may be necessary, though large-scale bilateral and multilateral global health initiatives such as the United States’ President's Emergency Plan for AIDS Relief and the Global Fund to Fight AIDS, Tuberculosis and Malaria (‘Global Fund’) have traditionally focused on vertically integrated disease intervention programs that provide funding to NGOs for implementation work, not HSS (13, 19). Though these global health initiatives have increasingly begun to recognize the need to support and implement HSS strategies, WHO's recommendation for 50% of international development assistance to be used for HSS may not have adequate support or a viable funding mechanism to address brain drain (4, 20, 21).

In attempting to address the challenges of brain drain, a number of strategies have been used in an attempt to expand HCW resource allocation and rebalance incentive structures. We examine strengths and weaknesses of these strategies and propose our own policy proposals to augment these efforts.

### Task shifting

Task shifting, involving the decentralization and delegation of tasks from healthcare professionals to less specialized health workers in order to expand the available human resource pool of the health workforce, has been identified as a potential tool in addressing brain drain (16, 22). This may involve the delegation of tasks from physicians to other healthcare providers or non-professional health workers, including nurses, nurse practitioners, community HCWs, and health worker volunteers. Task shifting can also be expanded to include delegation to family members and has been shown to have positive impact on health outcomes for diagnosis and treatment of HIV/AIDS (particularly in scaling-up of antiretroviral treatment), malaria, tuberculosis, and in the administration of immunizations in resource-poor countries while at the same time reducing costs (22–25).

Unfortunately, political and financial barriers, including needed investment, training, credentialing, health intervention protocols, regulatory and quality systems, and management structures to support task shifting, can place an untenable strain on already weak institutional capacity (22, 26). Most importantly, task shifting requires sustained medium and long-term investment necessary to enable comprehensive and integrated restructuring of healthcare teams, necessary adjustments to regulatory systems and HCW scope of practice, and development of training infrastructure and national government support of implementation of these programs (25, 26).

These commitments, to be sustaining, must also coincide with the overall strengthening of health systems, state ownership of the process, support from donor agencies, and utilize global guidelines to standardize and promote this training such as those developed by the WHO (22, 26, 27). Gains in task shifting may also be challenged by skill imbalances where the number of physicians may vastly exceed the number of available nurses, overall shortage of health workers who can adequately train and supervise newly trained workers, and inefficiency or poorer quality services that result from inoptimal task shifting (16). Further, HCW resistance to task shifting can also occur with physicians resisting task shifting and delegation of their duties, reluctance of HCWs to take on additional responsibilities without commensurate increase in remuneration, and professional councils and associations unsupportive of task shifting policies (23).

Importantly the use of task shifting, while potentially effective in training and deploying additional cadres of HCWs who can perform key public health functions, requires the same types of investments in the healthcare workforce that is currently absent and leads to brain drain. Without a viable and sustainable funding mechanism to support these efforts, potential gains from task shifting cannot be assured.

### Rebalancing incentives

Rebalancing of both financial and nonfinancial incentives is seen as imperative in addressing the microeconomic bases of HCWs external and internal brain drain, but these incentives alone cannot reverse this trend. Although countries like Malawi, Thailand and Ireland have had success in launching human resource programs for reversing brain drain by retooling incentive packages to offer increased research funding, monetary incentives, and offering other services and assistance, the sustainability of these gains is uncertain (1, 10, 28).

Financial incentives provide immediate results in retention and ability to hire new health workers, but require increased budgetary expenditures and investment that may result in other sacrifices in healthcare services and system building (19). Forms of financial incentives may include increased salary, allowances for housing or work shifts, benefit packages (such as pensions and retirement packages), access to loans or tax waivers, and education funding and training (19, 23, 29). These incentive packages are especially crucial in rural areas to attract and retain HCWs for underserved populations where working conditions may already be poor (23, 29).

Non-financial incentives, which seek to improve working conditions and social needs are also important, and when done in conjunction with financial incentives, can provide long-term improvement by enhancing worker morale, satisfaction and commitment (19, 29). Non-financial incentives are highly varied. These include improving working conditions, addressing social needs and professional development through better resources, occupational safety, manageable workload levels, management support and recognition, as well as flexible working schedules, access to training and career development, access to transportation and education for family members, and access to healthcare services (10, 19, 29).

However, the use of both requires strategic planning, investment and organizational modifications that must be carefully weighed against their potential positive/negative impact on retaining health workers (19, 29). For example, incentive programs must be judiciously crafted in order to ensure they are culturally-sensitive and are not perceived as unfair; otherwise, they may alienate HCWs leading to further loss of important human capital (19, 29). Importantly, incentives must be designed based on the specific needs of the health system, and require long-term political commitment, integration of efforts between public and private sectors (including national governments and NGOs), and monitoring and evaluation systems to determine impact (29). Optimally, planning for policy should involve the combination of both financial and non-financial incentives (29).

While restructuring incentives schemes can be effective in the short-term, they require significant investment and political commitment, may not be sustainable, and may not adequately address the root problems associated with brain drain. Hence, longer-term sustainability requires a more comprehensive, global governance approach.

### WHO Code

WHO has also attempted to address brain drain through policy. Most recently, the adopted WHO Global Code of Practice on International Recruitment of Health Personnel (‘WHO Code’) provides guidelines and attempts to establish a multilateral framework to addressing global HCW shortages (2, 30).

While a laudable first step, the WHO Code is international soft law and is non-binding (i.e. voluntary), has no enforcement mechanisms, and is confined in its formal implementation to member states, which may limit its scope and effectiveness. It also does not adequately address the current and past losses incurred by resource poor countries and the need for sustainable funding of HSS and HCW migration reforms encouraged by the WHO Code, which remains a continuing challenge (8, 11). Currently, very few high-income countries that are prime destinations for HCWs have made meaningful progress in implementing the WHO Code's recommendations into domestic policy (11).

In order to make the WHO Code more effective, an enabling governance structure supported by sustainable financing mechanisms to operationalize the Code should be explored.

## Policy proposal

Brain drain's complex global health equity issues cannot rely solely upon intermittent health infrastructure investment, task shifting, rebalancing of incentives, and voluntary efforts, unless they are supported by a sustainable global funding mechanism and a coordinated pathway for equitable HCW migration. In order to address these challenges, we propose a Global Health Resource Fund coordinated by WHO and the World Bank that will improve on existing efforts of identified strategies by creating new HCW incentives not reliant on national budgets or voluntary mechanisms.

### Global health resource fund

Cost-sharing and reimbursing resource-poor countries for brain drain losses should be considered as a viable option and has been encouraged as a potential solution by representatives of resource-poor countries impacted by brain drain (5, 9, 11, 28). Indeed, bilateral vehicles of direct reimbursement to resource-poor countries and other fee-based schemes have been proposed and recommended (5, 9, 11). However, bilateral agreements may fail to address the complex nature of the globalization of HCW migration involving a variety of different countries and entities including agencies of national governments, NGOs, private foundations and other actors.

We build upon these lessons by proposing a global fee-supported system similar to that employed by UNITAID for the sourcing of essential medicines. The Global Health Resource Fund (the ‘Fund’) would implement a dynamic fee structure assessed against a high-income country and private sector actors engaged in the recruitment of resource-poor country HCWs with collected funds earmarked for HSS and brain drain mitigation programs.

### Initial programmatic approach

In order to better mobilize support for implementation of this global health governance reform package, we would initially limit implementation of the fee-based structure to recruitment of HCWs who reside or who are trained in one of the 57 countries identified by WHO as having a critical HCW shortage. As these countries have the most urgent need, efforts must be taken to either discourage brain drain or provide some level of equitable compensation for their HCW losses, given the potential for adverse health impact. Inclusion in the system can easily be expanded to other countries identified with HCW shortages based on a sliding scale in future discussions.

The Fund would operate by establishing a baseline dues amount for any country or entity (e.g. healthcare facility, NGO, private foundation, development agency) that has engaged in recruitment and hire of an HCW from a critical shortage country. The proposed global fee would also be retrospective up to 5 years from implementation, weighted and adjusted based on the individual country factors (see Table 1).

**Table 1:** Global health resource fund fee system

Measurement of assessment	Description	Calculation	Benefits
Type and number of HCWs recruited	Calculation based upon type (e.g. physician, nurse, pharmacists, community healthcare worker) and number of HCW recruited from resource-poor country	Economic valuation of gained benefits similar to those that have been modelled before (e.g. medical education expenses, medical training infrastructure investment, loss of human capital/investment, economic gains realized by destination country)	Variable element of total fee weighted based on number and type of HCW recruited
Measure of proportionate impact of brain drain on country	Weighted measurement of proportionate severity of impact of brain drain	Based on percentage of HCWs lost per population/capita (e.g. this would allow for more equitable calculation of impact of HCW migration within subset of critical shortage countries)	Allows countries more severely impacted by brain drain to gain additional needed benefit
Measure of existing infrastructure and disease burden	Weighted assessment based on available domestic healthcare infrastructure capacity and disease burden	Independent assessment of healthcare infrastructure capacity (e.g. number of/accessibility of hospitals, clinics, community health services) and national health outcome data (e.g. infant/maternal mortality, immunization rates, DALYs)	Enables countries with lack of infrastructure and higher national disease burden to gain additional needed benefit

This assessment dynamically identifies resource-poor country emigration impact and developed country immigration benefits on an individual country basis. Global fees would be assessed upon commitment to hire a HCW and programmatically supported by domestic legislation to ensure collection and transfer of fees to the Fund.

Initially, retrospective fee assessments would be modelled upon listed factors (Table 1). These assessments would be refined using periodic projections of future HCW migration supported by a data collection mechanism (see below). Such forward-looking assessments would allow for better resource management and fund initial HSS and brain drain mitigation programs at the global, national, and local level. Critical shortage countries meeting a *de minimis* requirement (e.g. one health worker emigrating to a developed country annually) would also be entitled to a proportionate share of the funds to develop their own programs and offset their economic losses.

### Administration: WHO–World Bank

A new combined WHO–World Bank special agency would be created to administer the Fund, building upon existing efforts of the Health Systems Funding Platform (‘Platform’) currently consisting of the WHO, World Bank, the Global Fund, and the GAVI Alliance (21). Collectively, these entities are the largest funders of HSS and are engaged in efforts to develop harmonized joint system funding platforms for lowering transaction costs and increasing the efficiency of HSS (21).

Project financing would be supported and validated by a harmonized monitoring and evaluation framework. Financing would also be subject to periodic rigorous financial, accountability and program audits and would be available across stakeholder groups, including Ministries of Health, public–private partnerships, NGOs, and international organizations.

A WHO–World Bank agency would provide appropriate expertise for Fund administration and could work directly with the Platform to provide possible funding for Platform HSS projects, pool existing resources, and also work to provide technical guidance on implementation of other HCW initiatives such as the WHO Code (as well as earlier approaches, such as WHO Global Recommendation and Guidelines on Task Shifting) (27). This could include requiring all Fund and Platform partners, funding recipients, and contractors to adhere to the guidelines of the WHO Code, effectively changing the Code from voluntary to mandatory for participants who wish access to the Fund. The World Bank has a well-documented history of HSS projects, and WHO has clear subject matter expertise and a mandate to promote health equity goals and can also help in the implementation of WHO Code guidelines and recommendations (8).

### Governing board

To ensure accountability and transparency, a governing board, composed of representatives from critical shortage countries, other resource-poor countries, civil society, humanitarian aid organizations, medical societies, national health ministries, healthcare industry and providers, and NGOs, would oversee and keep the WHO–World Bank agency accountable. This stakeholder spectrum would ensure ethical and fair use of the funds, focusing on both resource-poor and individual HCW needs (31). Targeted health capacity and infrastructure gains through such a governance model could also have downstream benefits by helping to improve the delivery of services of other global health initiatives and also allow them to focus on disease treatment and intervention programs and delivery.

### Coordinated data collection and immigration pathways

Deincentivizing forms of HCW migration through a fee-based structure, however, may unfairly restrict movement of individuals seeking safety and financial autonomy (5). Thus, in conjunction with the Fund, a global framework for HCW migration should be developed to ensure efficient, humane and safe migration, as well as act as a data collection instrument for present and future resource analysis.

The WHO–World Bank agency would also oversee coordination and data capture by pulling together all possible data sources on HCW migration, including licensed health professional registration systems, HCW graduation listings, and data required to be reported by the WHO Code. It would work to establish harmonized HCW registration, licensing and migration systems in cooperation with national ministries of health and other regional actors using empirically based guidance to most effectively obtain, analyse, and provide feed back of this information.

To facilitate efficient migration, the agency could work in conjunction with the International Labour Organization (‘ILO’) to develop a database of healthcare employers and HCWs seeking migration opportunities, providing a single site for pre-qualified employers/employees, and promote additional data-driven capture and analysis. Both prospective employers and HCWs would be subject to a pre-qualification process and registration with the agency, with the employer paying a fee upon commitment to hire a candidate. The agency in cooperation with ILO could provide both parties with technical assistance and additional resources in order to ensure that migration is successful and necessary Fund fees (if applicable) are paid and collected. Such successful strategic ‘matching’ of applicants and employers through a single source or inter-country collaborations are well established in other systems of HCW placement, such as the National Resident Matching Program for post-graduate physician training in the United States, the Medical Education Partnership Initiative, and other partnerships (6, 32).

This approach could provide a safe, equitable, and efficient pathway for employers wishing to hire HCWs from abroad, and attract HCWs because participation can be coupled with mandates of important terms, including ethical treatment, commitment to hire at applicable professional skill level, and requiring employers to allow HCWs to return to source countries in a public health or other emergencies as suggested in previous proposals (11). Beyond an organized global system of HCW migration/matching and centralized collection of data, such a system would mitigate ‘brain waste’ by promoting entry into healthcare employment at relevant license and skill levels and on transparent terms of employment.

Costs could be relatively low, particularly if the program adopts a web or mobile-based platform. The Fund could provide initial funding, and/or public–private partnerships could help create model collaborations, systems, software, and setup for implementation. Health information technology grants through the Fund could also be provided to resource-poor settings to enable participation.

Furthermore, and crucial to the policy's success and implementation, there must be efforts at harmonization and mutual recognition of professional health qualifications/licensure at a regional and possibly global level similar to those taken in South East Asia programs (10). This approach will make emigration and immigration efforts less onerous, and may provide greater granularity as to healthcare and HCW country needs that allows more targeted interventions and policies. Coordination and policy coherence for this unique global health governance model on HCW migration could offset resource burdens of resource-poor countries while offering more efficient HCW migration systems for these workers and the entities in high-income countries that hire them.

## Conclusion

Brain drain from resource-poor countries continues; yet current attempts to address it remain insufficient largely due to the lack of sustainable funding, absence of binding rules on global HCW migration, and failure to adequately recompense origin countries for decades of human and economic losses from weakening of their national health systems. High-income countries and other actors should assume responsibility for the costs they have inflicted on these resource-poor populations. In response, they should provide equitable resource sharing to those countries most adversely impacted by HCW shortages and also work with the global community to develop a coordinated pathway to facilitate fair and equitable HCW migration.
